# A Novel Role of PP2A Methylation in the Regulation of Tight Junction Assembly and Integrity

**DOI:** 10.3389/fcell.2022.911279

**Published:** 2022-07-13

**Authors:** Diana Schuhmacher, Jean-Marie Sontag, Estelle Sontag

**Affiliations:** School of Biomedical Sciences and Pharmacy, University of Newcastle, Callaghan, NSW, Australia

**Keywords:** protein phosphatase 2A, methylation, tight junction, polarity, one-carbon metabolism

## Abstract

Tight junctions (TJs) are multiprotein complexes essential for cell polarity and the barrier function of epithelia. The major signaling molecule, protein serine/threonine phosphatase 2A (PP2A), interacts with the TJ and modulates the phosphorylation state of TJ proteins. An important PP2A regulatory mechanism involves leucine carboxyl methyltransferase-1 (LCMT1)-dependent methylation and protein phosphatase methylesterase-1 (PME1)-mediated demethylation of its catalytic subunit on Leu309. Here, using MDCK cells, we show that overexpression of LCMT1, which enhances cellular PP2A methylation, inhibits TJ formation, induces TJ ruffling, and decreases TJ barrier function. Conversely, overexpression of PME1 accelerates TJ assembly and enhances TJ barrier function. PME1-dependent PP2A demethylation increases during early Ca^2+^-dependent junctional assembly. Inhibition of endogenous PME1 delays the initial Ca^2+^-mediated redistribution of TJ proteins to cell-cell contacts and affects TJ morphology and barrier function. Manipulating one-carbon metabolism modulates TJ assembly, at least in part by affecting PP2A methylation state. The integrity of PP2A methylation is critical for proper targeting of PP2A to the TJ. It is necessary for PP2A complex formation with the TJ proteins, occludin and ZO-1, and proteins of the PAR complex, Par3 and atypical protein kinase C ζ (aPKCζ), which play a key role in development of cell polarity. Expression of a methylation incompetent PP2A mutant induces defects in TJ assembly and barrier function. aPKCζ-mediated Par3 phosphorylation is also required for targeting of the PP2A ABαC holoenzyme to the TJ. Our findings provide the first evidence for a role of LCMT1, PME1 and PP2A methylation/demethylation processes in modulating TJ assembly and functional integrity. They also position PP2A at the interface of one-carbon metabolism and the regulation of key TJ and polarity proteins that become deregulated in many human diseases.

## Introduction

The TJ, the most apical cell-cell junction, plays a critical role in epithelial tissue homeostasis by forming a paracellular permeability barrier (Monaco et al., 2021) and serving as a fence to maintain apicobasal cell polarity (Martin et al., 2021). The TJ is a multiprotein complex consisting of several integral membrane proteins (e.g., claudins, occludin, and junctional adhesion proteins), that interact with scaffolding proteins, such as ZO-1, a member of the zonula occludens protein family that plays an essential role in TJ assembly (Otani and Furuse, 2020; Rouaud et al., 2020). The intertwined network of TJ proteins is also linked to a variety of adaptor, cytoskeletal, polarity and signaling proteins. As such, the TJ plaque serves as a signaling platform that can influence cellular processes as diverse as cytoskeletal remodeling, gene expression, and cell polarity and division. Conversely, complex signaling events regulate TJ assembly, stability and function (Rusu and Georgiou, 2020). In vertebrate cells, the highly conserved, apical tripartite PAR polarity complex comprising Par3, Par6 and aPKCζ, is associated with the TJ; it is indispensable for TJ formation and establishment of apical polarization (Nagai-Tamai et al., 2002; Horikoshi et al., 2009). While TJ dysregulation is associated with numerous human diseases (Sawada, 2013), the intimate mechanisms underlying TJ formation and function are not fully elucidated (Otani and Furuse, 2020).

Highly regulated protein-protein interactions and phosphorylation/dephosphorylation events are essential for dynamic TJ assembly/disassembly (Van Itallie and Anderson, 2018; Rouaud et al., 2020). They modulate TJ barrier properties, affecting its degree of leakiness to selected solutes (Otani and Furuse, 2020; Monaco et al., 2021). They are also indispensable for formation of the PAR complex and development of apical polarization (Martin et al., 2021). For instance, aPKC-mediated phosphorylation of Par3 on Ser827 is required for apical domain development (Nagai-Tamai et al., 2002). Significantly, many TJ-associated proteins are phosphoproteins (Van Itallie and Anderson, 2018) that can be targeted for dephosphorylation by PP2A, a major Ser/Thr phosphatase and signaling molecule (Schuhmacher et al., 2019). PP2A catalytic activity negatively regulates epithelial TJ assembly in MDCK (Nunbhakdi-Craig et al., 2002) and Caco-2 (Seth et al., 2007) cells. PP2A co-immunoprecipitates with and dephosphorylates several TJ proteins, including occludin (Nunbhakdi-Craig et al., 2002; Seth et al., 2007), ZO-1 (Nunbhakdi-Craig et al., 2002), and JAM-A (Iden et al., 2012). In MDCK cells, PP2A forms a complex with and regulates aPKCζ (Nunbhakdi-Craig et al., 2002), a kinase essential for establishment and maturation of TJs in vertebrate epithelial cells (Iden et al., 2012) and cell polarization (Hong, 2018). In *Drosophila*, PP2A also associates with aPKC (Chabu and Doe, 2009), and binds to and dephosphorylates Par3 (Krahn et al., 2009) and Par6 (Ogawa et al., 2009), thereby antagonizing aPKC signaling and playing an essential role in cell polarization. Yet, how exactly PP2A regulates the mammalian PAR complex is unknown (Schuhmacher et al., 2019).

A majority of PP2A signaling molecules in mammalian cells are “ABC” heterotrimers made up of a catalytic C subunit (PP2Ac), a structural A subunit, and one of many regulatory “B” subunits belonging to four distinct (B, B′, B″ and B’’’) families (Schuhmacher et al., 2019). The association of a particular regulatory B subunit with the AC core enzyme influences PP2A subcellular distribution and binding to other proteins, and determines substrate specificity and selectivity. Of particular interest, pools of ABαC holoenzymes containing the regulatory Bα subunit (PP2A-Bα) are targeted to the TJ in MDCK cells where they regulate the phosphorylation state of TJ proteins (Nunbhakdi-Craig et al., 2002). PP2A regulation is complex and multifaceted, involving also posttranslational modifications of its constituent subunits and interactions with multiple scaffolding and regulatory proteins (Schuhmacher et al., 2019).

Reversible C-terminal methyl-esterification (methylation) of PP2Ac at the conserved Leu309 residue within the AC core enzyme is an important PP2A regulatory mechanism that controls formation of PP2A holoenzymes (Nasa and Kettenbach, 2020). PP2Ac methylation involves the sole action of the methyltransferase, LCMT1, and supply of the universal methyl donor, S-adenosylmethionine (SAM), which is governed by one-carbon metabolism (Skovierova et al., 2016). Numerous studies have shown that LCMT1-dependent methylation does not directly affect PP2Ac activity but differentially modulates the affinity of certain regulatory subunits for the AC core enzyme (Janssens et al., 2008). It is especially critical for recruiting PP2A-Bα and other B-type isoforms to AC dimers, while only facilitating or having no effect on binding of B′, B″ and B”’ subunits. As such, LCMT1 depletion is associated with a massive loss of PP2A-Bα, which becomes degraded when unbound to the methylated AC core enzyme (Lee and Pallas, 2007). In contrast to B subunits, B′, B″ and B”’ subunits are less sensitive to the lack of PP2Ac methylation (Janssens et al., 2008; Nasa and Kettenbach, 2020; Lyons et al., 2021). Thus, changes in PP2Ac methylation state can shift PP2A substrate specificity by tightly controlling the cellular repertoire of PP2A holoenzymes.

Removal of PP2Ac methyl group is carried out by the protein phosphatase methylesterase, PME1, and occurs in AC dimers, but not ABC heterotrimers (Tolstykh et al., 2000). Structural studies suggest that PME1 can also block PP2Ac activity by binding to its active site (Xing et al., 2008). However, cellular studies do not support the hypothesis that PME1 directly induces PP2A inactivation (Sents et al., 2013). Apart from its role as a methylesterase, PME1 may rather serve an important function in controlling cellular PP2A activity by associating with and stabilizing an inactive PP2A population (Ogris et al., 1999; Longin et al., 2008). The reactivation of these latent PP2Ac species involves the action of a specific PP2A activator and is tightly coupled to holoenzyme assembly (Sents et al., 2013). The association of LCMT1 with the PP2Ac active site within AC dimers facilitates the methylation reaction, thereby providing an exquisite mechanism for converting activated AC dimers into substrate-specific holoenzymes (Stanevich et al., 2014). The importance of proper regulation of PP2A holoenzyme assembly is illustrated by the link between changes in PP2A methylation and subunit composition with many human diseases, including cancer (Fowle et al., 2019) and neurodegenerative disorders (Sontag and Sontag, 2014).

Since methylation is especially critical for ABαC formation and stabilization (Tolstykh et al., 2000), and ABαC co-localizes and forms a complex with occludin and ZO-1 at the TJ (Nunbhakdi-Craig et al., 2002), we hypothesized that PP2Ac methylation is a key TJ regulatory mechanism. Here, using MDCK cells, we investigated the effects of perturbing endogenous PP2Ac methylation on the regulation of major TJ-associated and regulatory proteins, i.e., the transmembrane occludin, scaffolding ZO-1, and polarity Par3 proteins.

## Materials and Methods

### Reagents and Antibodies

Unless indicated, all chemicals and compounds [L-homocysteine (Hcy); homocysteine thiolactone (HTL); SAM; AMZ-30; ABL127; okadaic acid (OA)] were purchased from Sigma/Merck Millipore. Antibodies used in this study included: Mouse (clone 16B12, Covance) and rabbit (clone C29F4, Cell Signaling Technology) anti-HA; mouse anti-Myc (clone 9B11, Cell Signaling Technology); mouse anti-methyl PP2Ac (clone 2A10, Merck Millipore); mouse anti-demethyl-PP2Ac (clone 1D6; Merck Millipore); mouse anti-PP2Ac (#610556, BD Transduction); mouse anti-LCMT1 clone 4A4 (Merck Millipore); mouse anti-PME-1 (Sontag et al., 2007); rabbit (Nunbhakdi-Craig et al., 2002) and mouse (clone 2G9, Merck Millipore) anti-Bα; rabbit anti-Na^+^/K^+^ ATPase (#3010, Cell Signaling Technology); mouse anti-occludin (clone OC-3F10; Thermo Fisher Scientific); mouse anti-ZO-1 (clone ZO1-1A12, Thermo Fisher Scientific); rabbit anti-Par3 (#07-330, Merck-Millipore); rabbit anti-aPKCζ (sc-216, Santa Cruz Biotechnology); mouse (clone C4, Merck Millipore) and rabbit (#AAN01, Cytoskeleton Inc.) anti-actin; mouse anti-α-tubulin (clone DM1A; Sigma).

### Plasmids

Plasmids used in this study included: pBabe encoding hemagglutinin (HA)-tagged LCMT1 or myc-tagged PME1 (Sontag et al., 2007); pcDNA3.1 encoding HA-tagged PP2Ac or the L309Δ PP2Ac mutant (Sontag et al., 2007); pRcCMV encoding a kinase deficient mutant of aPKCζ (aPKCζ^mut^) (Sontag et al., 1997). pcMV-Tag3b (Agilent Technologies) encoding myc-tagged Par3 (Par3^WT^) or the Par3 S827A mutant (Par3^S827A^) were obtained after subcloning from corresponding SRHis-PAR-3 and SRHis-PAR-3 S827A plasmids (Nagai-Tamai et al., 2002). All plasmids were verified by sequencing.

### Cell Culture and Transfection

All experiments were performed in, and stable clones generated from, the highly polarized MDCK D5 clonal kidney cell line (Brewer and Roth, 1995), herein referred to as MDCK. Control and stable MDCK cell lines were maintained in 100-mm dishes in Dulbecco’s modified Eagle medium (DMEM, Thermo Fisher Scientific) containing 25 mM Hepes, pH 7.4, 10% fetal bovine serum (FBS, Bovogen Biologicals), and 10 μg/ml gentamycin (Thermo Fisher Scientific). Cells were transiently transfected with the indicated plasmids using METAFECTENE^®^ PRO reagent (Biontex Laboratories GmbH, Germany) at a 1:5 plasmid to reagent ratio. Cells mock-transfected with the empty vector (EV) were used as controls (Control MDCK) in all our experiments and behaved like untransfected cells (Nunbhakdi-Craig et al., 2002). Control and MDCK cells stably expressing HA-PP2Ac (MDCK-WTC) have been previously characterized (Nunbhakdi-Craig et al., 2002). MDCK cells stably expressing myc-Par3^WT^ (MDCK- Par3^WT^), myc-Par3^S827A^ (MDCK-Par3^S827A^), myc-PME1 (MDCK-PME1), HA-LCMT1 (MDCK-LCMT1), HA-L309Δ (MDCK-L309Δ), or aPKCζ^mut^ (MDCK-aPKCζ^mut^) were generated after transfection with the indicated plasmid and antibiotic selection (either 600 μg/ml G418 [Thermo Fisher Scientific], 200 μg/ml hygromycin [Roche] or 1 μg/ml puromycin [Sigma]). Transfected protein expression was boosted by incubating cells for 16 h with 1 mM sodium butyrate, and was systematically verified by immunoblotting and immunofluorescence (Nunbhakdi-Craig et al., 2002). We verified that similar results were obtained with distinct batches of each stable cell line generated (Nunbhakdi-Craig et al., 2002). To achieve complete polarization, cells were plated at confluency on polyester Transwell™ filters (0.4 μm; Corning) and grown for 4–5 days in cell culture medium (Nunbhakdi-Craig et al., 2002).

### Ca^2+^switch Experiments and Studies of Tight Junctions Assembly

In some experiments, cells were subjected to a Ca^2+^ switch. To induce TJ disassembly, confluent cell monolayers grown in normal 1.8 mM Ca^2+^ (NC)-containing cell culture medium were incubated overnight in low Ca^2+^ (LC) medium (Ca^2+^-free SMEM supplemented with 3 μM Ca^2+^, GlutaMax™, 5% dialysed FBS, and 10 μg/ml gentamycin; all reagents from Thermo Fisher Scientific). The Ca^2+^ switch was carried out by transferring cells for the indicated time from LC to NC medium, in the presence or absence of compounds, as specified in the figure legends. Of note, drug concentrations were adjusted to compensate for short-versus long-term incubations, to achieve maximal effects without inducing cytoxicity, based on preliminary experiments and our earlier work (Taleski et al., 2021). Transfected protein expression was induced by sodium butyrate prior to the Ca^2+^ switch (Nunbhakdi-Craig et al., 2002). In other experiments, cells were trypsinized, plated at confluency and cultured for 24 h in NC medium, which allows for complete TJ resealing (Nunbhakdi-Craig et al., 2002). Transfected protein expression was induced by sodium butyrate as soon as cells were attached.

### Transepithelial Resistance Measurements

For TER measurements, cells were plated at confluency in Transwell inserts and grown in NC medium. TER was measured in confluent cell monolayers subjected to a Ca^2+^ switch. In this case, rapid TJ disassembly, as assessed by obtaining background TER values, was induced by incubating confluent cell monolayers for 1 h in Ca^2+^-deprived LC medium containing 1 mM EGTA; cells were then switched back to NC medium for the indicated time, as described previously (Nunbhakdi-Craig et al., 2002). In other experiments, cells were plated at confluency in Transwell inserts and cultured for 72 h in NC medium to allow complete TJ maturation. Transfected protein expression was induced with sodium butyrate as soon as cells were attached. For each time point, TER was measured using an Epithelial Volt/Ohm Meter (EVOM^2^; World Precision Instruments). Particular care was taken to measure TER under strictly similar experimental conditions across cell lines (Sheller et al., 2017). TER values (Ω.cm^2^) were normalized to the filter area of the monolayer and calculated by subtracting blank values from the filter and bathing medium (Nunbhakdi-Craig et al., 2002).

### Confocal and Phase Contrast Microscopy

For most experiments, cells were grown on glass coverslips coated with poly-L-lysine (Merck Millipore; #P4707); when indicated, cells were grown on Transwell filters. To visualize TJ proteins and PP2A subunits, cells were fixed for 5 min at −20°C with absolute methanol (Nunbhakdi-Craig et al., 2002). To study the distribution of LCMT1 and PME1, cells were fixed for 20 min with 4% paraformaldehyde then permeabilized for 5 min with phosphate buffered saline (PBS; Thermo Fisher Scientific) containing 0.1% Triton X-100 and 1% bovine serum albumin (BSA). All cells were washed, blocked for 1 h in PBS containing 3% BSA, and sequentially incubated for 1 h with the indicated primary antibody followed by Alexa Fluor^488^ or Alexa Fluor^594^-conjugated secondary antibodies (#A11001, #A11005, #A11012, #27034, Thermo Fisher Scientific). The samples were mounted using DAPI fluoromount G (ProSciTech) and examined on a Nikon D-Eclipse C1 confocal microscope using a ×60 objective. Captured images (5 x-y or x-z stacks) were exported to NIH ImageJ. Quantification of TJ ruffling was performed in ZO-1 labeled cells by dividing the actual junction length by the distance between tricellular junctions (Lynn et al., 2020). Cells were also examined by phase contrast microscopy on a Zeiss Axiovert 200 microscope, after replacing the normal cell culture medium with phenol red-free DMEM (Thermo Fisher Scientific) (Nunbhakdi-Craig et al., 2003). All images were transferred to Adobe Photoshop/Illustrator 2021 (Adobe Systems Incorporated) for figure preparation.

### Cell Lysis, Detergent Extraction and Immunoprecipitation

After washing with PBS, total cell homogenates (1 × 100-mm dish/condition) were prepared in 400 μL buffer 1 (10 mM Tris, pH 7.4, 150 mM NaCl, 1 mM dithiothreitol, 0.5 μM OA, 5 mM PMSF, 1% NP-40, Sigma Protease Inhibitor Cocktail™, and Sigma Phosphatase Inhibitor Cocktail™) using a motor pestle. For analyses of PP2A methylation state, 1 μM ABL-127 was added in the buffer to prevent demethylation. To prepare detergent-insoluble fractions, total cell lysates were further centrifuged for 90 min at 20,000 x g to generate NP-40 detergent-soluble (supernatant) and -insoluble (pellet) fractions, which were resuspended in buffer 1 (Taleski et al., 2021). Aliquots of total homogenates and detergent-insoluble fractions were sonicated and cleared by centrifugation at 13,000 x g for 3 min at 4°C. The protein concentration was determined using the Bradford protein assay kit (Bio-Rad). For immunoprecipitation assays, total cell lysates (1 × 100-mm dish/condition) were prepared in 400 μl buffer 2 (Buffer 1 + 0.5% sodium deoxycholate, without dithiothreitol). A 50 μl aliquot of the total lysates was set aside, and the remaining fraction immunoprecipitated overnight at 4°C with either anti-HA (clone C29F4; Cell Signaling Technology #11846), or -myc (clone 9B11; Cell Signaling Technology #5698S) antibody coupled magnetic beads. Magnetic beads were washed 5 times in buffer 2 before being resuspended in gel loading buffer. Aliquots of total lysates and corresponding immunoprecipitates were analyzed in parallel by Western blot.

### Gel Electrophoresis and Western Blotting

Protein samples (∼50 μg/lane or equivalent volumes) were resolved on NuPAGE 4%–12% Bis-Tris gels (Thermo Fisher Scientific), with pre-stained protein standards (BioRad) used as molecular weight markers (Taleski et al., 2021). In most cases, blots were cut horizontally between molecular weight markers to allow simultaneous immunostaining and reprobing of the top and bottom parts with distinct validated antibody species (Taleski et al., 2021). Western blot analyses were performed using the specified primary antibodies, followed by Infrared IRDye®-labelled secondary antibodies, and visualized using the Odyssey Infrared imaging system (LI-COR Biosciences). Band intensity was determined using the associated Image Studio Lite, version 5.2.5 Software (LI-COR Biosciences) to quantify protein expression levels. Anti-actin/tubulin antibodies were used to normalize for protein loading. PP2A methylation state was determined as described previously (Sontag et al., 2013), using antibodies recognizing methylated or demethylated PP2Ac, and after normalizing for total PP2Ac. For blots in Figures 6B, 7C–E, immunoreactive proteins were detected using SuperSignal Chemiluminescence substrates (Pierce Chemical Co.,), as described previously (Nunbhakdi-Craig et al., 2003).

### Statistical Analyses

Data were analyzed for normal distribution and statistical significance using GraphPad Prism 9. Data with *p* < 0.05 were considered significant.

## Results

### LCMT1 Delays While PME1 Accelerates the Initial Redistribution of ZO-1, Occludin and Par3 to Cell-Cell Contacts During Ca^2+^-Induced TJ Assembly

**FIGURE 1:**
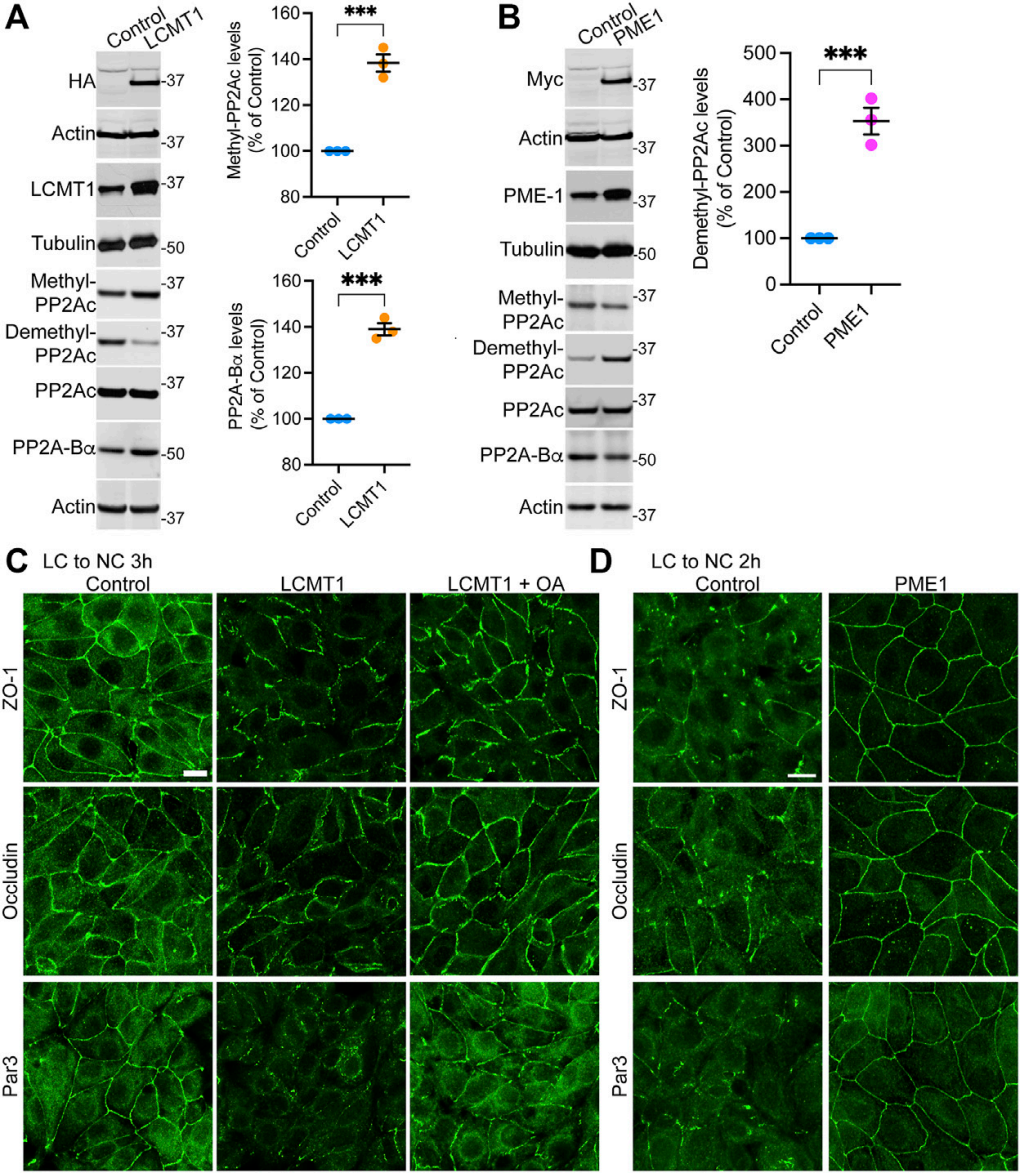
Overexpression of LCMT1 and PME1 in MDCK cells affects the initial redistribution of ZO-1, occludin and Par3 to cell-cell contacts during Ca^2+^-induced TJ reassembly. **(A)** Total lysates from MDCK-LCMT1 and control MDCK cells were analyzed by Western blot (left) for HA-LCMT1, LCMT1, PP2A-Bα, and total/methylated/demethylated PP2Ac levels. Relative expression levels (right) of PP2A-Bα and methylated PP2Ac were quantified in these cells (mean ± SEM; *n* = 3; Student’s *t*-test; ***p < 0.001). **(B)** Representative immunoblots of myc-PME1, PME1, PP2A-Bα, and total/methylated/demethylated PP2Ac levels in total lysates from MDCK-PME1 and control MDCK cells (left). The relative levels of demethylated PP2Ac (mean ± SEM; *n* = 3; Student’s *t*-test; ***p < 0.001) were quantified in these cells (right). **(C)** Distribution of ZO-1, occludin and Par3 in control MDCK and MDCK-LCMT1 cells 3 h after a Ca^2+^ switch. A subset of MDCK-LCMT1 cells was incubated with 100 nM OA (+OA) during the switch. **(D)** Distribution of ZO-1, occludin and Par3 in control MDCK and MDCK-PME1 cells switched for 2 h from LC to NC medium. For **(C–D)**, representative images from three separate experiments are shown. Scale bars = 10 μm.

To assess the regulatory role of PP2A methylation/demethylation in TJ assembly, we first generated MDCK cells stably expressing HA-tagged LCMT1 (MDCK-LCMT1) or Myc-tagged PME1 (MDCK-PME1). Confocal analyses of these stable MDCK cell lines (Supplemental Figure S1) showed that both LCMT1 and PME1 were diffusely distributed throughout the cytoplasm of cells grown at low density; PME1 was enriched in the nucleus, as observed in other cells (Longin et al., 2008). The methyltransferase and methylesterase were primarily cytoplasmic and absent from intercellular junctions in confluent MDCK monolayers. Western blot analyses confirmed that overexpression of LCMT1 in MDCK cells augmented endogenous methylated PP2Ac levels while expectedly decreasing demethylated PP2Ac amounts (Figure 1A). In agreement with earlier studies in N2a cells (Sontag et al., 2007), higher PP2Ac methylation in MDCK-LCMT1 cells was accompanied with increased PP2A-Βα protein expression levels, likely due to enhanced formation and stabilization of methylated ABαC trimers (Tolstykh et al., 2000). Conversely, overexpression of PME1 increased basal amounts of demethylated PP2Ac in MDCK cells (Figure 1B); this was associated with lower levels of methylated PP2Ac and PP2A-Bα, as reported in other cells (Sontag et al., 2007; Longin et al., 2008).

To determine how LCMT1/PME1 modulate TJ formation, cells were subjected to a Ca^2+^ switch. In this widely used assay, cell-cell contacts are completely disrupted by incubating confluent cells under low Ca^2+^ conditions (LC medium), which results in solubilization and redistribution of TJ proteins to intracellular compartments. Re-addition of Ca^2+^ by switching cells to NC medium induces the progressive *de novo* formation and maturation of intercellular junctions. As expected (Nunbhakdi-Craig et al., 2002), a large proportion of cytoplasmic ZO-1, occludin and Par3 was already recruited to cell-cell borders in control MDCK cells switched for 3 h from LC to NC medium (Figure 1C). However, overexpression of LCMT1 significantly delayed this initial redistribution, as evidenced by the more disrupted staining pattern of all three proteins at areas of cell-cell contact 3 h after the Ca^2+^ switch. Interestingly, these inhibitory effects were partially reversed by incubating MDCK-LCMT1 cells with okadaic acid (OA), a PP2A inhibitor that accelerates TJ formation in MDCK cells (Nunbhakdi-Craig et al., 2002). In contrast to LCMT1, overexpression of PME1 in MDCK cells markedly accelerated the recruitment of ZO-1, occludin and Par3 to cell-cell contacts. While switching control MDCK cells for 2 h from LC to NC medium was insufficient to induce a complete redistribution of ZO-1, occludin and Par3 to cell-cell borders, these proteins were fully localized at intercellular junctions in MDCK-PME1 cells 2 h after the Ca^2+^ switch (Figure 1D).

### Endogenous Demethylated PP2Ac Levels Increase During Initial Ca^2+^-Induced Cell-Cell Contact Formation

**FIGURE 2:**
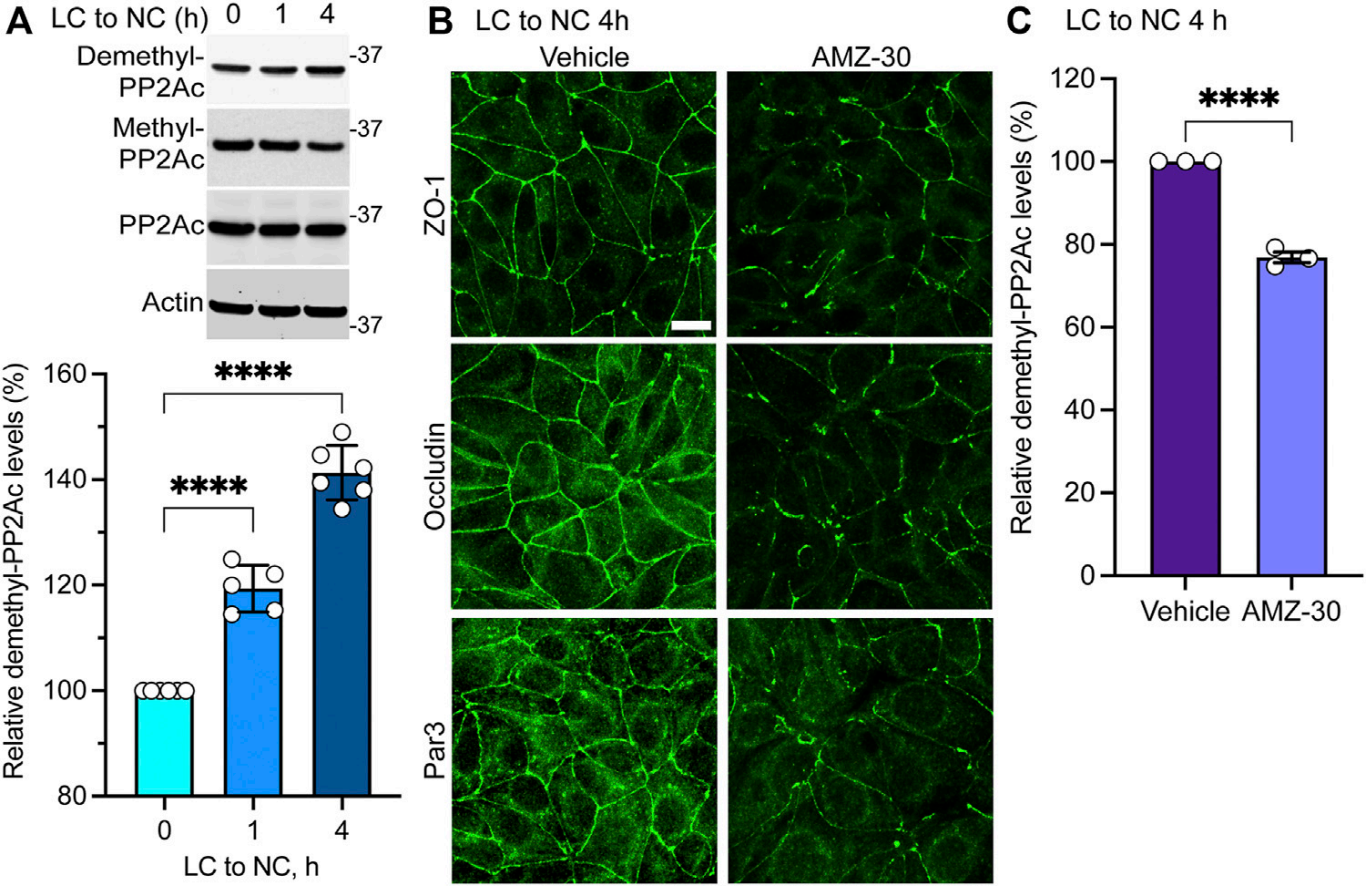
Endogenous PME1-mediated PP2A demethylation is enhanced during early Ca^2+^-induced TJ formation in MDCK cells. **(A)** Endogenous demethylated PP2Ac levels were analyzed by immunoblotting (top) and quantified (bottom) in MDCK cells switched for 0, 1 and 4 h from LC to NC medium. Data (mean ± SD) were appraised using one-way ANOVA (F = 168.8; *p* < 0.0001) with post hoc Dunnett’s multiple comparisons test. ****p < 0.0001. **(B)** Representative distribution of ZO-1, occludin and Par3 in MDCK cells incubated for 4 h with either 20 μM AMZ-30 or vehicle during a Ca^2+^ switch. Similar results were obtained in three separate experiments. Scale bar = 10 μm. **(C)** Relative levels of demethylated PP2Ac were quantified in these cells (mean ± SEM; *n* = 3; Student’s *t*-test; ****p < 0.0001).

Results obtained in MDCK-PME1 cells suggest that enhanced PME1 activity plays a positive role during the early steps of junctional assembly. In support of this hypothesis, Western blot analyses of untransfected MDCK cells revealed that there was a progressive accumulation of endogenous demethylated PP2Ac during the initial phases of Ca^2+^-induced cell-cell contact formation (Figure 2A). Maximal demethylation of PP2Ac in MDCK cells correlated with the near complete redistribution of ZO-1, occludin and Par3 at cell-cell borders 4 h after the Ca^2+^ switch (Figure 2B). In contrast, incubation of untransfected MDCK cells with the specific PME1 inhibitor, AMZ-30, hindered the recruitment of these proteins to cell-cell contacts under the same conditions. AMZ-30 effectively prevents PME1-mediated PP2Ac demethylation, subsequently causing an indirect build-up of methylated PP2Ac (Bachovchin et al., 2010; Taleski et al., 2021). Accordingly, the delayed junctional redistribution of TJ proteins correlated with a reduction in demethylated PP2Ac levels in AMZ-30-, relative to vehicle-treated MDCK cells (Figure 2C).

Altogether, our findings suggest that increased PME1-dependent PP2A demethylation is required for the initial Ca^2+^-mediated redistribution of TJ proteins to cell-cell contacts in MDCK cells. Conversely, enhancing LCMT1-mediated PP2A methylation inhibits this process.

### LCMT1 Induces the Appearance of Ruffled Tight Junctions

**FIGURE 3:**
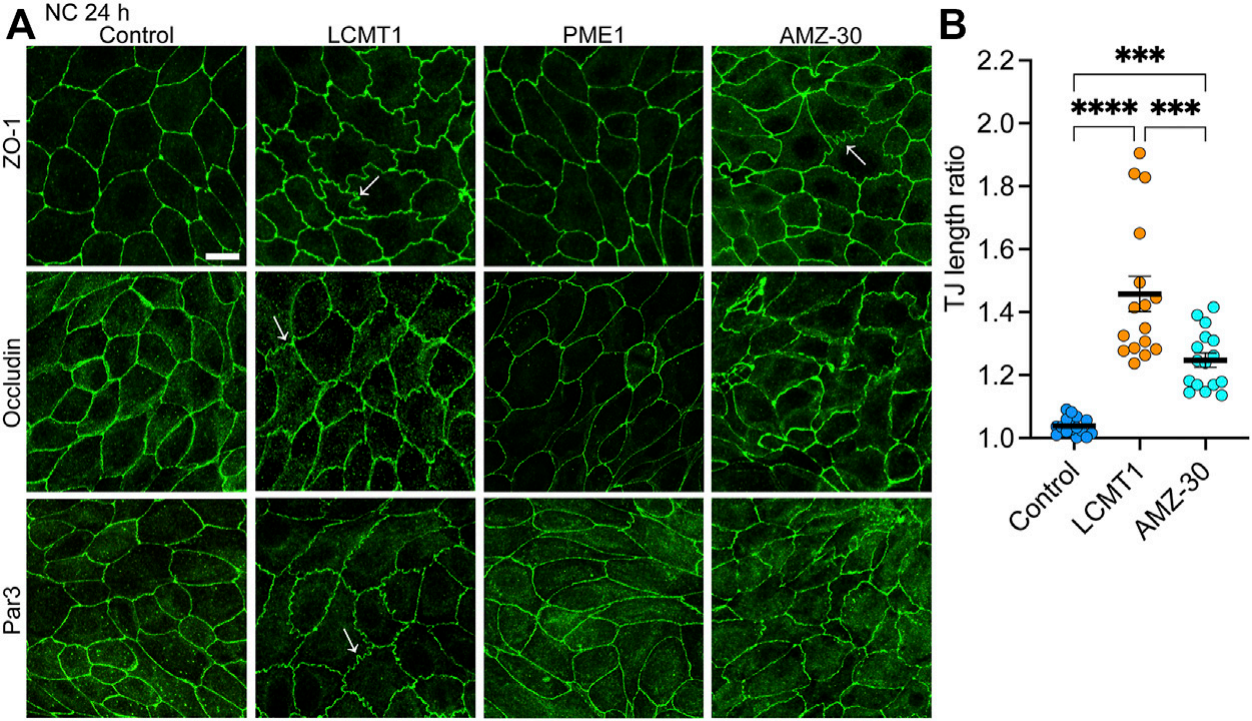
Overexpression of LCMT1 and inhibition of PME1 induce TJ ruffling in MDCK cells. **(A)** Comparative distribution of ZO-1, occludin and Par3 in control MDCK, MDCK-LCMT1, and MDCK-PME1 cells cultured for 24 h in NC medium. A subset of control cells was incubated in the presence of 1 μM AMZ-30 (AMZ-30). Arrows indicate examples of ruffled TJs. Representative images from three separate experiments are shown. Scale bar = 10 μm. **(B)** Quantification of TJ ruffling was performed by dividing the actual junction length by the distance between tricellular junctions. Data (mean ± SEM) were appraised using one-way ANOVA (F = 35,26; *p* < 0.0001) with Šídák’s multiple comparisons test. ****p < 0.0001; ***p < 0.001.

We next investigated the distribution of TJ proteins in our stable cell lines cultured for 24 h in NC medium, which allows for complete TJ resealing and stabilization (Nunbhakdi-Craig et al., 2002). As expected, under these conditions, ZO-1, occludin and Par3 had a typical cobblestone-like junctional organization in control MDCK cells (Figure 3A). Their distribution was similar in MDCK-PME1 cells. While LCMT1 delayed the initial recruitment of ZO-1, occludin and Par3 to cell-cell contacts during junctional assembly (Figure 1C), those proteins were ultimately localized at cell-cell borders in MDCK-LCMT1 cells cultured for 24 h in NC medium. However, nearly all MDCK-LCMT1 cells displayed TJs with a striking zigzaggy, ruffled morphology rather than the primarily continuous linear appearance of normal TJs in control MDCK cells. Such TJ ruffling has been linked to changes in TJ morphology under a variety of conditions (Lynn et al., 2020). Some TJ ruffles and irregularities in TJ protein distribution were also observed in MDCK cells cultured for 24 h in NC medium containing AMZ-30. However, there was less ruffling in these cells than in LCMT1-overexpressing cells (Figure 3B). Thus, enhancing PP2A methylation (overexpression of LCMT1) or blocking endogenous PP2A demethylation (inhibiting PME1) induce TJ morphological changes.

### LCMT1 Inhibits the Development of Tight Junctions Barrier Function, Which Is Enhanced by PME1

**FIGURE 4:**
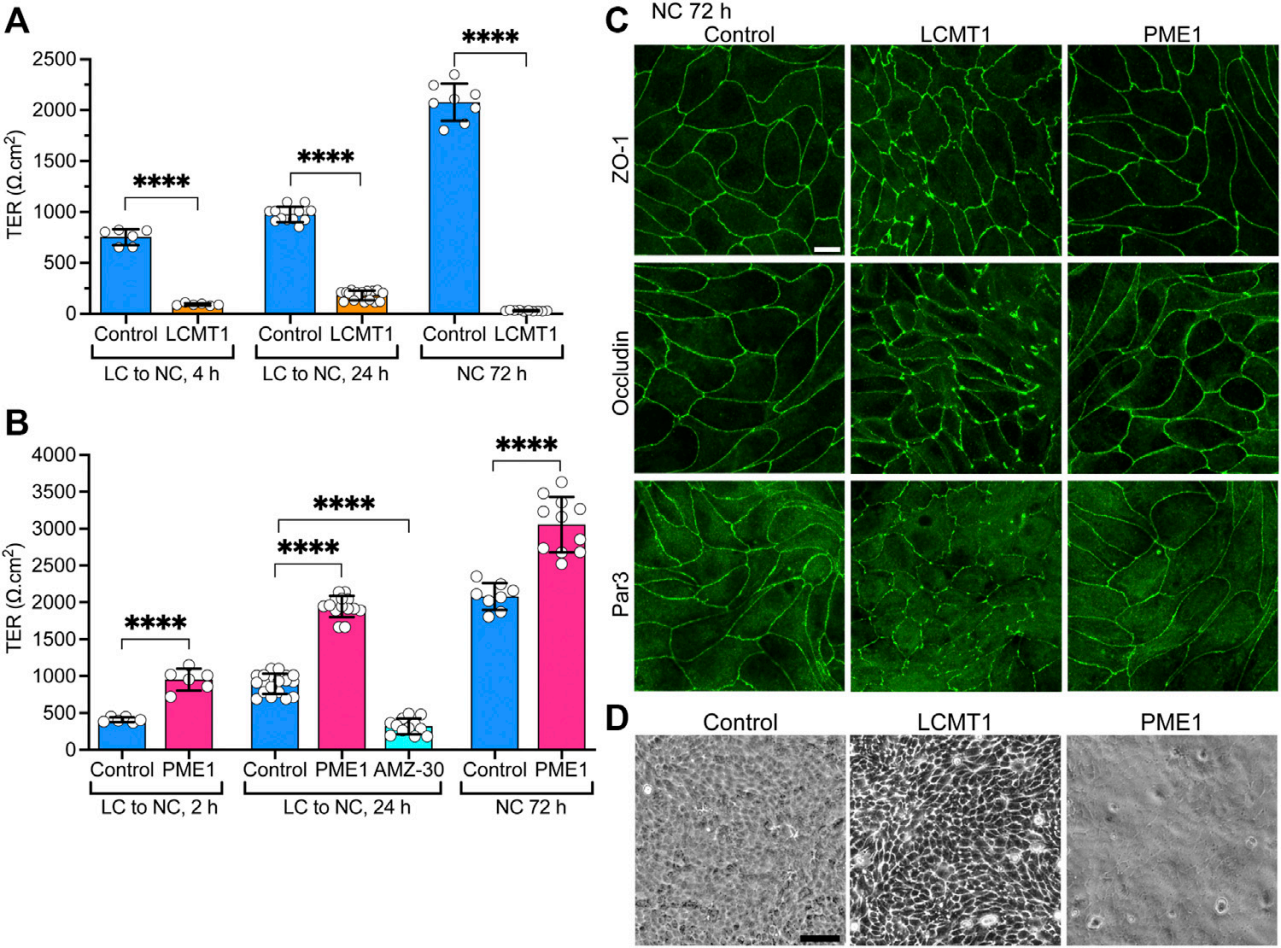
Differential effects of LCMT1 and PME1 on TJ morphology and barrier function in MDCK cells. **(A–B)** TER values were measured in control MDCK **(A–B)**, MDCK-LCMT1 **(A)** and MDCK-PME1 **(B)** cells at either 2, 4 or 24 h after a Ca^2+^switch, and 72 h after culture in NC medium. A subset of control cells was treated with 1 μM AMZ-30 (AMZ-30). Values (mean ± SD from individual Transwells from separate experiments) were assessed using one-way ANOVA [F = 788, *p* < 0.0001 for **(A)**; F = 292.3, *p* < 0.0001 for **(B)**] with Šídák’s multiple comparisons test; ****p < 0.0001. **(C)** Comparative distribution of ZO-1, occludin and Par3 in control MDCK, MDCK-LCMT1, and MDCK-PME1 cells cultured for 72 h in NC medium. **(D)** Analysis of confluent control MDCK, MDCK-LCMT1, and MDCK-PME1 monolayers by phase-contrast microscopy (×10 objective). For **(C–D)**, representative images from three separate experiments are shown. Scale bars = 10 μm.

Since LCMT1 and PME1 can modulate TJ assembly, and proper TJ formation is essential for the integrity of TJ barrier function (Monaco et al., 2021), we further assessed whether these enzymes affect the development of TJ barrier function. Measuring TER is widely used to assess dynamic changes in TJ barrier function (Anderson and Van Itallie, 2009; Srinivasan et al., 2015). In agreement with the early inhibitory effect of LCMT1 on TJ formation (Figure 1C), TER values were much lower in MDCK-LCMT1 than in control MDCK cells 4 h after the Ca^2+^ switch (Figure 4A). MDCK-LCMT1 cells still exhibited lower TER values than controls 24 h after the Ca^2+^ switch, a time point where TJs are normally completely resealed (Nunbhakdi-Craig et al., 2002). When cell monolayers were cultured for 72 h in NC medium, which normally allows for complete MDCK cell polarization (Nunbhakdi-Craig et al., 2002), MDCK-LCMT1 monolayers had strikingly lower TER values than polarized control cells, suggesting marked defects in TJ barrier function. TER values were significantly higher in MDCK-PME1 cells than in control cells 2 h after the Ca^2+^ switch (Figure 4B), in support of its positive role in early TJ assembly (Figure 1D). MDCK-PME1 cells also developed higher TER than control cells 24 h or 72 h after culture in NC medium. Furthermore, AMZ-30 mediated PME1 inhibition decreased TER values in MDCK cells, supporting the hypothesis that PME1 enhances TJ barrier function.

Differences in the TER of MDCK-LCMT1 at 72 h compared to 24 h (Figure 4A) suggested that prolonged expression of LCMT1 worsens defects in TJ barrier function. This prompted us to comparatively examine the expression levels and distribution of ZO-1, occludin and Par3 in our cells. Similar ZO-1, occludin and Par3 protein expression levels were observed in control MDCK, MDCK-LCMT1 and MDCK-PME1 cells cultured for 24 and 72 h in NC medium (Supplemental Figure S2). Further examination of these cells showed a similar junctional localization of ZO-1, occludin and Par3 in control MDCK and MDCK-PME1 cells cultured for 72 h in NC medium (Figure 4C), as was observed after 24 h (Figure 3A). In contrast, the pattern of TJ protein distribution was much more disrupted in MDCK-LCMT1 cells at 72 h compared to 24 h. Abundant TJ ruffles but continuous cell-cell border labeling were the hallmarks of MDCK-LCMT1 cells cultured for 24 h in NC medium (Figure 3A). However, in addition to wavy TJs, many areas of discontinuous ZO1, occludin and Par-3 staining became apparent in these cells after 72 h in culture (Figure 4C), suggesting that altered TJ protein distribution could underlie the drop in TER observed in these cells. Further qualitative analysis of our cell lines by phase contrast microscopy revealed the presence of visibly enlarged intercellular gaps in MDCK-LCMT1 monolayers, compared to control MDCK cells (Figure 4D). By comparison, MDCK-PME1 cells formed very tight monolayers.

Altogether, our findings indicate that altering LCMT1, PME1, and PP2A methylation can induce significant effects on TJ assembly, morphology and barrier function.

### Metabolites of the Methylation Cycle can Affect Tight Junctions Assembly by Modulating PP2A Methylation

**FIGURE 5:**
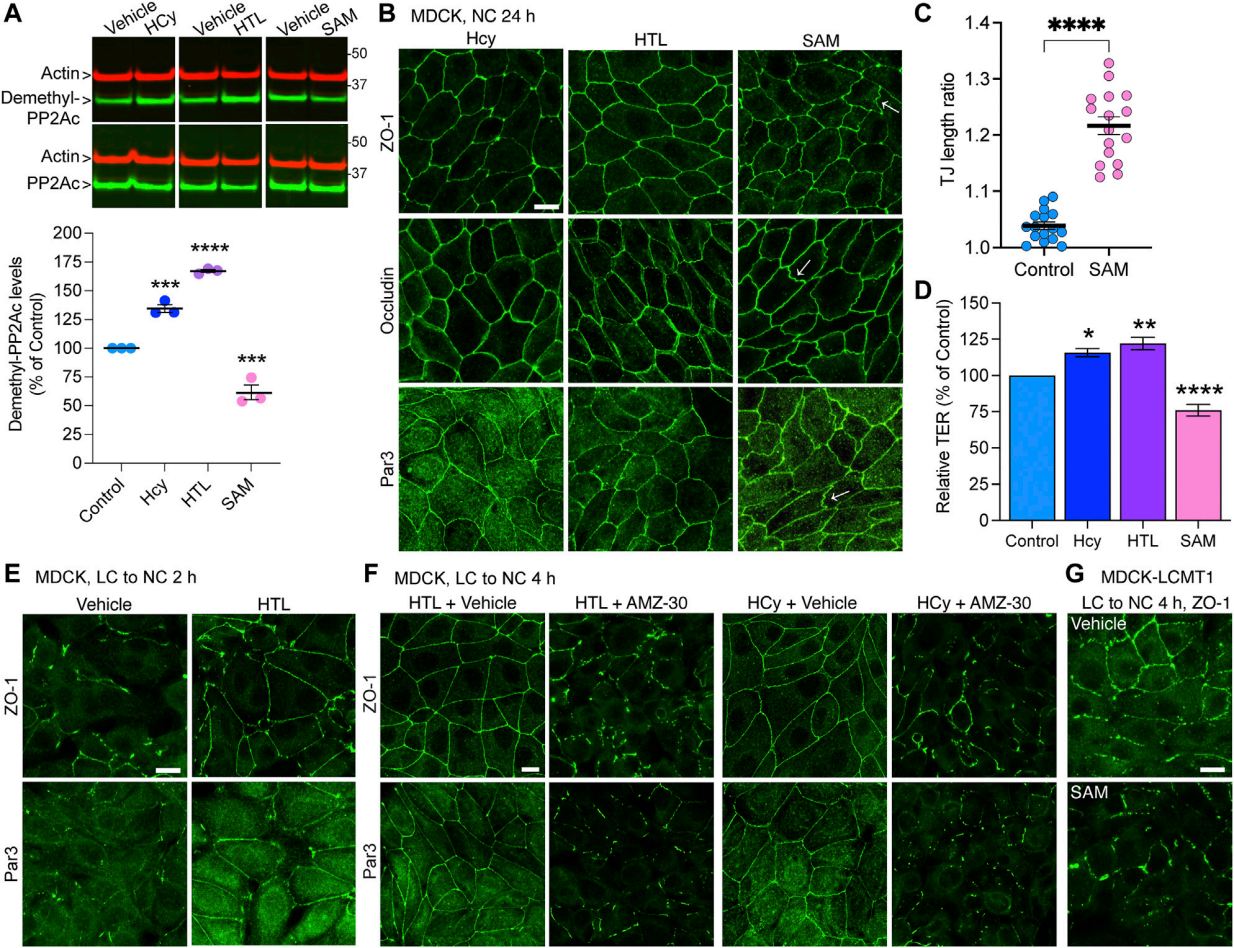
Hcy, HTL and SAM metabolites modulate PP2A methylation state and TJ assembly. **(A)** Total and demethylated PP2Ac were analyzed by Western blot (top) in total lysates from MDCK cells incubated for 24 h in NC medium in the presence of either 100 μM Hcy, 100 μM HTL, 100 μM SAM, or vehicle. Demethylated PP2Ac levels were quantified in these cells (bottom). Data (mean ± SEM; *n* = 3) were appraised using one-way ANOVA (F = 150.6; *p* < 0.0001) with post hoc Dunnett’s test. ****p < 0.0001, ***p < 0.001, versus vehicle-treated controls. **(B)** Distribution of ZO-1, occludin and Par3 in these cells. Arrows indicate examples of ruffled TJs. **(C)** Quantification of TJ ruffling in SAM-treated MDCK cells (mean ± SEM; Student’s *t*-test; ****p < 0.0001). **(D)** TER values were measured in cells from **(B)**. Data (mean ± SEM; *n* = 6 Transwells) were analyzed using one-way ANOVA (F = 42.12; *p* < 0.0001) with post hoc Dunnett’s multiple comparisons test. ****p < 0.0001, ***p = 0.001, *p < 0.05, versus vehicle-treated control cells. **(E)** Distribution of ZO-1 and Par3 in MDCK cells switched for 2 h from LC to NC medium in the presence of either 200 μM HTL or vehicle. **(F)** Distribution of ZO-1 and Par3 in MDCK cells switched for 4 h from LC to NC medium containing either 200 μM Hcy or HTL, together with 20 μM AMZ-30 or vehicle. **(G)** Distribution of ZO-1 in MDCK-LCMT1 cells switched for 4 h from LC to NC medium in the presence of either 200 μM SAM or vehicle. For **(B,E–G)**, representative confocal images are shown; similar results were obtained in three separate experiments. Scale bars = 10 μm.

Notably, PP2A methylation is dependent on the provision of methyl groups, which is linked to the methylation cycle of one-carbon metabolism (Ducker and Rabinowitz, 2017). We have previously shown that incubating N2a cells with the methyl donor, SAM, boosts LCMT1-dependent PP2Ac methylation (Sontag et al., 2007). In contrast, disturbances in one-carbon metabolism can lead to abnormal elevation of homocysteine (Hcy) and stimulate the formation of S-adenosylhomocysteine, a potent allosteric inhibitor of mammalian methyltransferases (Ducker and Rabinowitz, 2017). Indeed, we have reported that elevated levels of either homocysteine (Hcy) or its derived metabolite, homocysteine-thiolactone (HTL) (Skovierova et al., 2016) promote the accumulation of demethylated PP2Ac in N2a cells and *in vivo* (Sontag et al., 2007; Taleski et al., 2021). Based on the TJ regulatory role of PP2A methylation, we used similar experimental conditions to investigate the effects of these key metabolites on TJ assembly in MDCK cells. In agreement with our studies in N2a cells (Sontag et al., 2007; Taleski et al., 2021), treatment of MDCK cells with Hcy/HTL increased, while incubation with SAM decreased endogenous demethylated PP2Ac levels (Figure 5A). Treatment with SAM induced the formation of TJ ruffles (Figures 5B,C), and decreased TER values in MDCK cells cultured for 24 h in NC medium (Figure 5D); those effects were reminiscent of those of AMZ-30. As observed earlier with PME1, incubation with Hcy/HTL had no apparent effect on the junctional distribution of ZO-1, occludin and Par3 (Figure 5B) but increased TER values (Figure 5D) in MDCK cells cultured for 24 h in NC medium. We next investigated the effects of these metabolites during Ca^2+^-mediated junctional reassembly, focusing on ZO-1 and Par3. Treatment with HTL mimicked the effects of PME1 (Figure 1D) and accelerated the early recruitment of ZO-1 and Par3 to cell-cell contacts during initial Ca^2+^-mediated TJ reassembly (Figure 5E); of note, we used here HTL rather than Hcy because it was faster acting. Co-treatment with AMZ-30 hindered the recruitment of ZO-1 and Par3 to cell-cell contacts in MDCK cells incubated with Hcy/HTL (Figure 5F). Incubation with SAM further inhibited the junctional redistribution of ZO-1 in MDCK-LCMT1 cells during a Ca^2+^ switch (Figure 5G).

Thus, our results uncover a novel regulatory link between key compounds of one-carbon metabolism, PP2A methylation state and Ca^2+^-dependent TJ assembly.

### Expression of a Methylation-Incompetent PP2Ac Mutant Induces Defects in Tight Junctions Assembly and Barrier Function

To further demonstrate the importance of dynamic PP2A methylation/demethylation processes in TJ regulation, we generated MDCK-L309Δ cells stably expressing the HA-tagged L309Δ PP2Ac mutant, in which the Leu309 methylation site is deleted. Significantly, expressing the L309Δ mutant mimics the effects of reducing LCMT1 expression on methylation-dependent holoenzyme assembly (Lyons et al., 2021). MDCK-L309Δ cells were comparatively analyzed with previously characterized control MDCK and MDCK-WTC cells stably expressing HA-tagged PP2Ac (Nunbhakdi-Craig et al., 2002). Of note, due to autoregulation of PP2Ac expression levels, transfected PP2Ac species can only be marginally overexpressed (Baharians and Schonthal, 1998). Thus, ∼half of endogenous PP2Ac was replaced by methylation-competent WTC in MDCK-WTC cells (Sontag et al., 2007) or methylation-incompetent L309Δ in MDCK-L309Δ cells.

Phase contrast analyses showed that expression of both WTC and L309Δ altered the normal morphology of MDCK cells (Supplementary Figure S3). While growing control MDCK cells typically formed compact cell colonies, dividing MDCK-WTC cells were elongated and had numerous protrusions reminiscent of a fibroblast-like phenotype. Growing MDCK-L309Δ cells also failed to form organized islets; they generally appeared larger and flatter than MDCK-WTC or control cells. At confluency, all cell lines formed cobblestone-like monolayers, albeit differences in cell size and morphology were visible.

**FIGURE 6:**
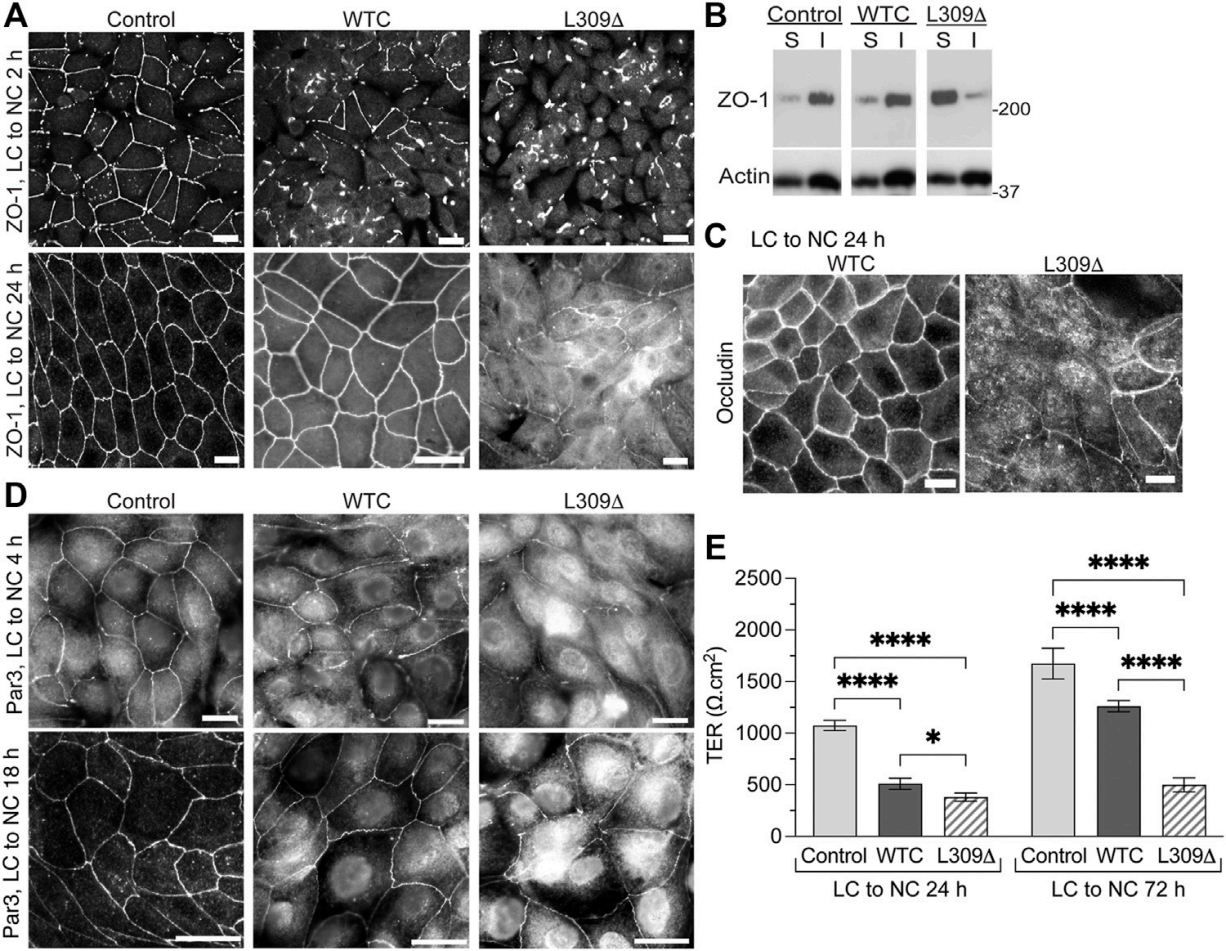
Expression of the L309Δ mutant in MDCK cells induces defects in TJ assembly and barrier function. **(A)** Distribution of ZO-1 in control MDCK, MDCK-WTC and MDCK-L309Δ cells 2 h or 24 after being switched from LC to NC medium. **(B)** Western blot analyses of ZO-1 in detergent-soluble (S) and -insoluble (I) fractions prepared from these cells 24 h after the Ca^2+^ switch. **(C)** Distribution of occludin in MDCK-WTC and MDCK-L309Δ cells 24 h after the Ca^2+^ switch. **(D)** Distribution of Par3 in control MDCK, MDCK-WTC and MDCK-L309Δ cells 4 h or 18 h after being switched from LC to NC medium. For **(A–D)**, similar results were found in three separate experiments. Scale bars = 10 μm. **(E)** TER values (mean ± SD; *n* = 8) were measured in control MDCK, MDCK-WTC and MDCK-L309Δ cells 24 and 72 h after the Ca^2+^ switch. Data were appraised with two-way ANOVA (cell line: F = 519.9, *p* < 0.0001; effect of time: F = 357.7, *p* < 0.0001; interaction: F = 56.3, *p* < 0.0001) with Tukey’s multiple comparisons test. ****p < 0.0001; *p < 0.05.

We have previously reported that expression of WTC in MDCK cells increases PP2Ac activity by ∼30%, which significantly delays but ultimately does not prevent the redistribution of ZO-1 to cell-cell junctions during TJ formation (Nunbhakdi-Craig et al., 2002). As observed with WTC, expression of L309Δ inhibited the early recruitment of ZO-1 to cell-cell borders during Ca^2+^-induced TJ reassembly (Figure 6A). When cells were switched for 24 h from LC to NC medium, ZO-1 had a typical chicken-wire junctional organization in both control and MDCK-WTC cells, although residual cytoplasmic ZO-1 staining can be seen in MDCK-WTC cells (Nunbhakdi-Craig et al., 2002). Visible defects in ZO-1 protein distribution, including interrupted junctional staining and pronounced cytosolic accumulation, were observed in MDCK-L309Δ cells under the same conditions.

Incorporation of proteins into newly formed membrane junctional complexes correlates with enhanced resistance to extraction with nonionic detergents (Stuart and Nigam, 1995; Farshori and Kachar, 1999). Western blot analyses confirmed that ZO-1 was primarily resistant to detergent extraction in control MDCK cells 24 h after the Ca^2+^ switch (Figure 6B). Likewise, a major proportion of ZO-1 was found in detergent-insoluble fractions prepared from MDCK-WTC cells, albeit a small increase in ZO-1 solubility occurs in these cells (Nunbhakdi-Craig et al., 2002). In contrast, most cellular ZO-1 was partitioned in the soluble fraction of MDCK-L309Δ cells.

As observed with ZO-1, occludin was ultimately distributed at intercellular junctions in MDCK-WTC cells (Nunbhakdi-Craig et al., 2002), but cytosolic accumulation of occludin and discontinuous occludin labeling at areas of cell-cell contact were visible in MDCK-L309Δ cells 24 h after the Ca^2+^ switch (Figure 6C). Consistent with the negative role of WTC in TJ formation in MDCK cells (Nunbhakdi-Craig et al., 2002), WTC also delayed the translocation of Par3 to cell-cell borders upon Ca^2+^-induced junctional assembly (Figure 6D). Again, marked abnormalities in the distribution of endogenous Par3 were detected in MDCK-L309Δ cells subjected to the Ca^2+^ switch.

We have previously reported that expression of WTC promotes TJ leakiness by inducing the dephosphorylation of key TJ-associated proteins, an effect that was reversed by OA (Nunbhakdi-Craig et al., 2002). Accordingly, TER values were lower in MDCK-WTC than control MDCK cells 24 h or 72 h after the Ca^2+^ switch (Figure 6E). However, L309Δ-mediated defects in TJ assembly were associated with a more pronounced decrease in the TER of MDCK-L309Δ, compared to control MDCK and MDCK-WTC cells; this effect was especially emphasized at 72 h. These findings suggest that irreversibly deregulating PP2A holoenzyme assembly by expressing a methylation-ablating mutation of PP2Ac interferes with proper TJ assembly.

### The Integrity of PP2A Methylation is Required for Proper Targeting of PP2A to the Tight Junctions and its Association with Tight Junctions Proteins and Par3

**FIGURE 7:**
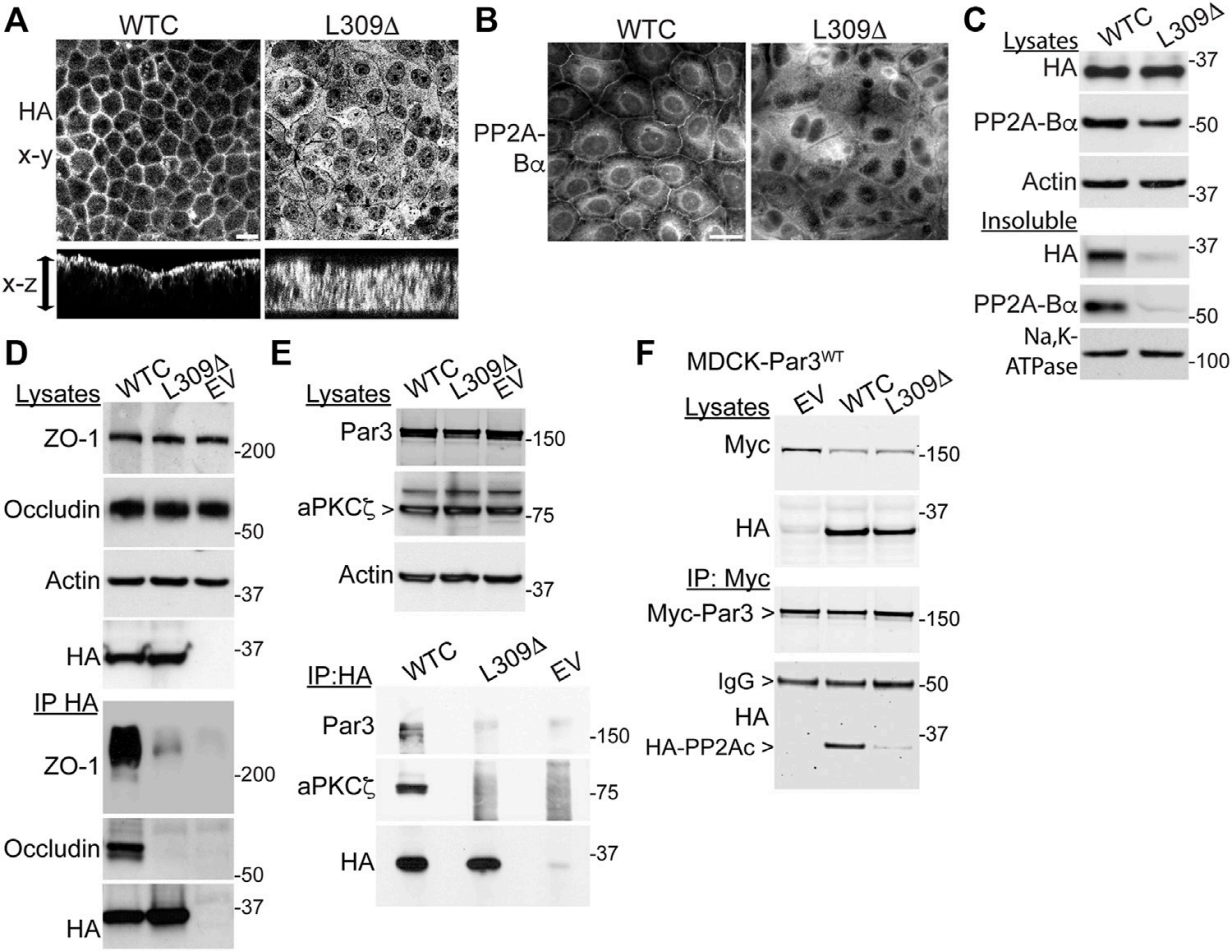
Expression of the L309Δ mutant in MDCK cells alters PP2A distribution and complex formation with TJ proteins and Par3. **(A)** Polarized MDCK monolayers were analyzed by confocal microscopy for the distribution of WTC and L309Δ using anti-HA antibodies. Representative apical x-y and transversal x-z sections are shown; the arrow indicates the monolayer thickness. **(B)** Representative distribution of PP2A-Bα in MDCK-WTC and MDCK-L309Δ cells 20 h after a Ca^2+^ switch. **(C)** Western blot analysis of HA-PP2Ac and PP2A-Bα in total lysates and NP40-detergent-insoluble fractions prepared from MDCK-WTC and MDCK-L309Δ cells cultured in NC medium. Sodium potassium pump (Na,K-ATPase), membrane marker. **(D)** Western blot analyses of ZO-1 and occludin in total lysates and HA-immunoprecipitates (IP) prepared from MDCK cells transfected with WTC, L309Δ, or empty vector (EV). **(E)** Western blot analyses of endogenous Par3 and aPKCζ in total lysates and HA immunoprecipitates (IP) prepared from WTC-, L309Δ- or EV-, transfected MDCK cells. **(F)** Western blot analyses of myc-Par3 and HA-PP2Ac in total lysates and corresponding myc immunoprecipitates (IP) prepared from MDCK-Par3 cells stably expressing myc-Par3, that were transfected with either WTC, L309Δ, or EV. IgG, immunoglobulin. For **(A–F)**, similar results were obtained in three separate experiments. Scale bars = 10 μm.

Since LCMT1-dependent PP2Ac methylation is especially critical for assembly of the ABαC isoforms (Nasa and Kettenbach, 2020) that associate with the TJ (Nunbhakdi-Craig et al., 2002), we hypothesized that L309Δ-induced TJ defects could result from improper targeting of PP2A to the TJ. Analysis of polarized cell monolayers by confocal microscopy first showed that, in contrast to WTC (Nunbhakdi-Craig et al., 2002), the L309Δ mutant was absent from apical cell-cell junctions and rather diffusely distributed throughout the cytoplasm (Figure 7A). Antibodies against PP2A-Bα were next used to assess the distribution of ABαC, since it is known that PP2A-Bα subunits become degraded when not incorporated into the methylated holoenzyme complex (Lee and Pallas, 2007; Lyons et al., 2021). As observed in MDCK cells (Nunbhakdi-Craig et al., 2002), pools of PP2A-Bα were present at cell-cell junctions in MDCK-WTC cells 20 h after a Ca^2+^ switch (Figure 7B). In contrast, PP2A-Bα was essentially undetectable at cell-cell borders in MDCK-L309Δ cells. By comparison, pools of PP2A-Bα were still clearly localized at cell-cell contacts in MDCK-LCMT1, MDCK-PME1 and MDCK cells treated with either Hcy/HTL or SAM (Supplemental Figure S4). Western blot analyses of total cell lysates confirmed that the L309Δ mutant was expressed at similar levels as WTC in our stable cell lines (Figure 7C). However, total PP2A-Bα expression levels were decreased in MDCK-L309Δ, relative to MDCK-WTC cells. In agreement with ABαC being enriched with TJ proteins in detergent-insoluble fractions from MDCK cells (Nunbhakdi-Craig et al., 2002), both detergent-insoluble pools of WTC and PP2A-Bα were present in MDCK-WTC cells. However, L309Δ and PP2A-Bα were hardly detected in detergent-insoluble fractions from MDCK-L309Δ cells, in line with the confocal distribution data.

The improper recruitment of PP2A to cell-cell borders in MDCK-L309Δ cells could also compromise its normal interaction with the multiprotein TJ complex (Nunbhakdi-Craig et al., 2002). Indeed, immunoprecipitation assays carried out in these cells further revealed that, in contrast to WTC (Nunbhakdi-Craig et al., 2002), the L309Δ mutant failed to associate with ZO-1 and occludin (Figure 7D). In MDCK cells, PP2A also co-immunoprecipitates with aPKCζ (Nunbhakdi-Craig et al., 2002), a kinase that associates with Par3 in a phosphorylation-dependent manner (Nagai-Tamai et al., 2002). PP2A-Par3 interactions have been described in *Drosophila* (Krahn et al., 2009). Based on these findings and the deregulation of Par3 distribution in MDCK-L309Δ cells, we also investigated whether PP2Ac can co-immunoprecipitate with Par3 in a methylation-dependent manner. Like aPKCζ, Par3 was readily detected in HA-immunoprecipitates prepared from MDCK-WTC, but not MDCK-L309Δ or control cells (Figure 7E). Conversely, significant amounts of WTC, but not L309Δ, were found in myc immunoprecipitates prepared from MDCK cells stably expressing myc-tagged Par3 (Figure 7F).

Altogether, our results indicate that formation of a complex between PP2A and key TJ and polarity proteins is methylation-dependent.

### Targeting of ABαC to Cell-Cell Junctions Depends on aPKCζ-Dependent Phosphorylation of Par3 at Ser827

**FIGURE 8:**
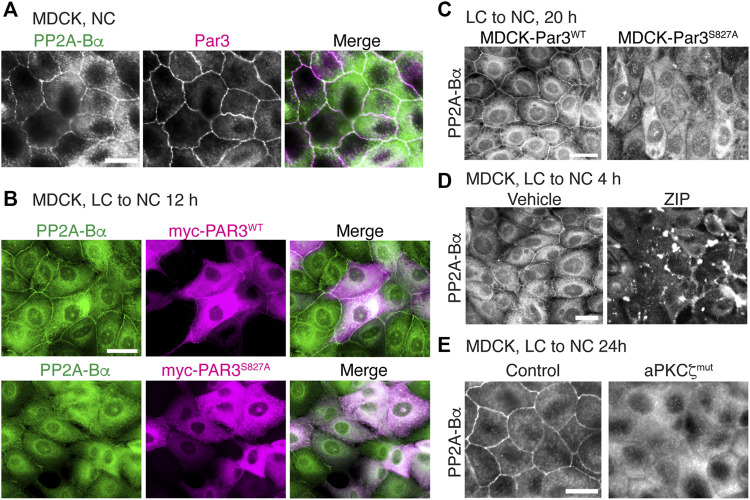
Interfering with aPKCζ-dependent phosphorylation of Par3 affects the targeting of ABαC to the TJ. **(A)** Co-localization of endogenous PP2A-Bα and Par3 in confluent MDCK cells labeled with anti-Bα and anti-Par3 antibodies. **(B)** Comparative distribution of PP2A-Bα in MDCK cells transiently transfected with Par3^WT^ or the Par3^S827A^ mutant, 12 h after a Ca^2+^ switch. Note that PP2A-Bα is present at the junctions between cells expressing Par3^WT^, but not Par3^S827A^. **(C)** Comparative distribution of PP2A-Bα in stable MDCK-Par3^WT^ and MDCK-Par3^S827A^ cells, 20 h after a Ca^2+^ switch. **(D)** Distribution of PP2A-Bα in MDCK cells switched for 4 h from LC to NC medium in the presence of 50 μM ZIP or vehicle. **(E)** PP2A-Bα is distributed at cell-cell junctions in control MDCK, but not in MDCK-aPKCζ^mut^ cells, 24 h after a Ca^2+^ switch. For **(A–E)**, representative images from three separate experiments are shown. Scale bars = 10 μm.

In addition to forming a complex with methylated PP2A enzymes (Figures 7E,F), we also observed that Par3 co-localized with ABαC at cell-cell junctions in MDCK cells (Figure 8A). Notably, aPKC-induced phosphorylation of Par3 at the evolutionarily conserved Ser827 site is essential for cell-cell contact induced epithelial polarity development in MDCK cells (Nagai-Tamai et al., 2002) and other models (Martin et al., 2021). Expression of the Par3^S827A^ mutant, but not Par3^WT^, induces deleterious effects on the redistribution of ZO-1 to cell-cell borders during Ca^2+^-induced TJ assembly (Nagai-Tamai et al., 2002). These observations prompted us to test the hypothesis that perturbing the regulation of Par3 also influences the targeting of ABαC to intercellular junctions. PP2A-Bα was normally redistributed at cell-cell junctions in MDCK cells transiently transfected with Par3^WT^ (Figure 8B) and in stable MDCK-Par3^WT^ cells (Figure 8C) that were switched from LC to NC medium. However, it was primarily concentrated in the cytoplasm and essentially absent from areas of cell-cell contact in MDCK cells transiently (Figure 8B) or stably (Figure 8C) expressing Par3^S827A^. Moreover, incubation of MDCK cells with the widely used myristoylated aPKCζ pseudosubstrate inhibitor (ZIP), which interferes with aPKCζ activation (Tsai et al., 2015), prevented the sorting of PP2A-Bα to cell-cell junctions during TJ assembly (Figure 8D). While PP2A-Bα was normally redistributed at cell-cell contacts in control MDCK cells 24 h after the Ca^2+^ switch, it remained concentrated in the cytoplasm and was essentially absent from cell-cell borders in MDCK-aPKCζ^mut^ cells stably expressing a kinase deficient mutant of aPKCζ (Figure 8E). Thus, aPKCζ-mediated Par3 phosphorylation may be required for proper targeting of ABαC holoenzymes to the TJ.

## Discussion

Reversible protein phosphorylation is an essential, yet incompletely understood mechanism that regulates the formation and function of TJ and polarity complexes (Van Itallie and Anderson, 2018; Martin et al., 2021). Dynamic and concerted changes in protein kinase/phosphatase activities are required to fine tune TJ regulation to meet cellular needs and respond to rapid fluctuations in the extracellular environment. Several studies have established that increased activity of PP2A, a major cellular Ser/Thr phosphatase, negatively regulates TJ assembly (Nunbhakdi-Craig et al., 2002; Seth et al., 2007; Iden et al., 2012). Here, we provide the first evidence that PP2A methylation state, under the control of LCMT1, PME1 and one-carbon metabolism, critically modulates TJ assembly, morphology and barrier function. Of note, we found that knockdown of either LCMT1, PME1, PP2Ac or PP2A-Bα in MDCK cells promoted cell death, as reported in other cells (Lee and Pallas, 2007); thus, we used other approaches to modulate PP2A methylation and assess its role in TJ assembly.

Since PP2A activity controls nearly all cellular processes, tight control of PP2A substrate specificity is paramount for homeostasis. It largely depends on a panoply of regulatory “B” subunits that compete for binding to preassembled AC dimers (Schuhmacher et al., 2019). By differentially modulating the coupling of “B” subunits to the core enzyme, LCMT1-dependent PP2Ac methylation plays a critical role in controlling the biogenesis of substrate-selective PP2A holoenzymes. It is especially important for formation and stabilization of ABαC holoenzymes (Sents et al., 2013). Accordingly, we found that overexpressing LCMT1 in MDCK cells increased methylated PP2Ac and ABαC levels. The presence of higher quantities of methylated holoenzymes likely explains why Ca^2+^-mediated TJ assembly is inhibited in MDCK-LCMT1 cells, since enhancing PP2A activity also delays TJ formation in MDCK cells (Nunbhakdi-Craig et al., 2002). The inhibitory effects of LCMT1 were reversed by incubating cells with OA; this toxin not only inhibits PP2Ac activity to accelerate TJ assembly (Nunbhakdi-Craig et al., 2002; Seth et al., 2007), but also prevents LCMT1-dependent PP2A methylation (Lee and Stock, 1993).

Upon completion of cell-cell junction reassembly, we observed the striking formation of TJ ruffles in MDCK-LCMT1 cells. Those were not visible in MDCK-WTC cells, wherein net PP2Ac activity, but not methylation, is enhanced. TJ ruffling was also present, albeit to a lesser extent, when shifting the cellular balance towards enhanced PP2A methylation *via* treatment of MDCK cells with SAM or AMZ-30. The lesser effects of SAM and AMZ-30 are likely explained by different outcomes on cellular levels of methylated PP2A. Incubation of MDCK cells with SAM boosts endogenous LCMT1 activity levels. AMZ-30 inhibits PME1, but has no effect on LCMT1, so it only indirectly increases the ratio of methylated/demethylated PP2Ac in MDCK cells. Due to elevated basal levels of PP2Ac methylation in mammalian cells, endogenous PP2Ac methylation can only be enhanced by a small margin by SAM (Sontag et al., 2007) or AMZ-30. In contrast, higher cellular amounts of LCMT1 have the potential to amplify *de novo* formation and stabilization of methylated holoenzymes. Interestingly, TJ ruffling involves changes in the interaction of claudins with the ZO-1 scaffolding protein, which crosslinks transmembrane claudins to the actin cytoskeleton. These alterations lead to actin remodeling and subsequent changes in TJ morphology (Lynn et al., 2020). Numerous claudin isoforms form the paracellular permeability barrier (Monaco et al., 2021). It is postulated that changes in claudin composition serve to modulate TJ assembly and barrier function in response to various stimuli (Lynn et al., 2020). Due to the lack of suitable antibodies, we were unable to assess whether LCMT1 specifically enhances the expression levels of claudin-2, which is intimately linked to TJ ruffling (Lynn et al., 2020).

Since TJ ruffling can increase TJ paracellular permeability, it could underlie the decreased TER of MDCK-LCMT1 cells; yet, it is not always the case (Lynn et al., 2020). Moreover, prolonged LCMT1 expression ultimately led to TJ disruption, suggesting that additional mechanisms are at play. While overexpressed LCMT1 had no apparent effects on cellular amounts of ZO-1 and occludin, it could alter the protein expression levels of claudins and other TJ proteins not studied here, that can modulate TJ barrier function (Monaco et al., 2021). Furthermore, enhancing PP2A activity at the TJ induces the Ser/Thr dephosphorylation of TJ-associated phosphoproteins, including ZO-1, occludin and claudin-1, thereby enhancing TJ paracellular permeability (Nunbhakdi-Craig et al., 2002). Likewise, increased PP2A-mediated dephosphorylation of occludin at Thr residues stimulates TJ disassembly (Seth et al., 2007). TJ-associated ABαC holoenzymes are appropriately localized to dephosphorylate TJ proteins (Nunbhakdi-Craig et al., 2002), and pools of ABαC were expectedly present at cell-cell junctions in MDCK-LCMT1 cells. Thus, enhanced LCMT1-mediated ABαC formation and stabilization could also alter TJ barrier function by shifting the balance towards increased ABαC-mediated dephosphorylation of key TJ-associated proteins. Since complex epitope-specific phosphorylation events exert positive or negative effects on the localization, protein interactions and function of TJ proteins (Van Itallie and Anderson, 2018), phosphoproteomic studies will be needed to determine how exactly modulating PP2A methylation influences TJ protein phosphorylation state.

In line with the critical role of PP2Ac methylation on ABαC holoenzyme assembly, reduced LCMT1 expression induces a pronounced loss of PP2A-Bα (Lee and Pallas, 2007; Sontag et al., 2008). This effect can be recapitulated by overexpressing the methylation-ablating L309Δ mutant (Lyons et al., 2021). Accordingly, PP2A-Bα expression levels were significantly reduced in MDCK-L309Δ cells. Notably, B subunits are important for directing PP2A to specific subcellular locations and regulating its interaction with other proteins. In MDCK cells, the ABαC heterotrimer is targeted to the TJ where it forms a complex with occludin and ZO-1 (Nunbhakdi-Craig et al., 2002). Unlike methylation-competent WTC, the unmethylated L309Δ mutant is unable to form a complex with PP2A-Bα (Janssens et al., 2008). In contrast to WTC, it was not distributed at the TJ and failed to interact with occludin and ZO-1. There was also a substantial loss of ABαC at cell-cell borders in MDCK-L309Δ cells, relative to MDCK-WTC cells, in line with previous findings showing a net reduction of membrane-associated ABαC in L309Δ-transfected N2a cells (Sontag et al., 2013). The junctional loss of ABαC in MDCK-L309Δ cells was associated with defects in the redistribution of TJ proteins during TJ assembly and decreased TER, suggesting that proper targeting of PP2A to and association with the multiprotein TJ complex are important for TJ integrity. In support of this hypothesis, a similar loss of ABαC and TJ leakiness are observed in MDCK cells expressing SV40 small t; this viral antigen specifically forms a complex with PP2A AC dimers and displaces PP2A-Bα from ABαC trimers (Nunbhakdi-Craig et al., 2003). Deregulation of PP2A by SV40 small t also leads to actin reorganization in MDCK cells (Nunbhakdi-Craig et al., 2003). PP2A methylation modulates actin dynamics in N2a cells (Taleski et al., 2021). Thus, remodeling of the actin cytoskeleton could be an additional mechanism by which altered PP2A methylation induces defects in TJ assembly (Schuhmacher et al., 2019).

In contrast to LCMT1, PME1 positively regulated TJ assembly. Demethylated PP2Ac accumulated during the early stages of Ca^2+^-mediated TJ reassembly in MDCK cells. Overexpression of PME1 further increased demethylated PP2Ac levels and accelerated the redistribution of TJ proteins to cell-cell contacts during Ca^2+^-induced junctional formation. These effects were reminiscent of those of OA, which stimulates TJ formation in MDCK cells (Nunbhakdi-Craig et al., 2002), and promotes cellular PP2Ac demethylation (Favre et al., 1997). Conversely, inhibiting endogenous PME1 delayed TJ assembly. It is noteworthy that PME1 demethylates PP2Ac within AC dimers, but not methylated “ABC” heterotrimers (Tolstykh et al., 2000). Thus, enhancing PME1 activity is expected to increase cellular pools of demethylated AC dimers, which in turn can support biogenesis of methylation-insensitive, versus methylation-dependent holoenzymes. Resulting alterations in PP2A subunit composition impact substrate dephosphorylation, since different PP2A species oppose the action of different classes of kinases and display site-specific substrate preferences (Kruse et al., 2020). Albeit controversial (Sents et al., 2013), interaction of PME1 with PP2A holoenzymes could also lead to PP2A inactivation by a mechanism separate from demethylation (Xing et al., 2008). Interestingly, PME1 activation can be triggered by a rise in intracellular Ca^2+^ in other experimental models (Lee et al., 2018). Since there is a high basal level of PP2A activity and methylation in mammalian cells, Ca^2+^-induced PME1 activation provides a plausible mechanism for transiently altering endogenous PP2A substrate specificity during Ca^2+^-mediated TJ assembly. This would facilitate Ser/Thr kinase-dependent phosphorylation of TJ proteins, such as occludin, that occurs during TJ formation (Dorfel and Huber, 2012).

Importantly, the effects of PME1 must be limited in scope and restricted to specific localized pools of PP2A enzymes, since complete PP2A inactivation (Strack et al., 2004) and lack of methylation (Lee and Pallas, 2007) induce cell death. In support of this hypothesis, previous studies have shown that, surprisingly, overexpression of PME1 does not induce major detrimental consequences on total PP2Ac activity and cell functioning despite increasing PP2Ac demethylation (Longin et al., 2008). Thus, the lack of PP2Ac methylation and concomitant loss of ABαC must attain a certain threshold to start inducing marked cellular effects (Longin et al., 2008), such as the TJ defects observed in MDCK-L309Δ cells. In contrast to MDCK-L309Δ cells, pools of ABαC were still detected at cell-cell junctions in MDCK-PME1 cells, likely because preassembled ABαC heterotrimers are protected from demethylation by PME1 (Tolstykh et al., 2000). TJ proteins were also normally expressed and distributed in MDCK-PME1 monolayers. Thus, PME1 may function to enhance TJ stability and increase TER by promoting localized alterations in Ser/Thr phosphorylation of key TJ-associated proteins, without dramatically affecting TJ structure. Due to the intricacy of PP2A regulation, comprehensive studies will be required to fully elucidate the mechanisms underlying the differential TJ regulatory role of LCMT1, PME1 and L309Δ.

We also provide the first evidence that PP2A can form a complex with and regulate the distribution of Par3 in MDCK cells in a methylation-dependent manner. Notably, the correct subcellular localization of Par3 is crucial for its ability to direct cell polarization (Martin et al., 2021). Studies in *Drosophila* have shown that PP2A can bind to and dephosphorylate the fly homolog of Par3 at a conserved Ser residue; the importance of this mechanism is illustrated by the link between PP2A dysregulation and polarity defects (Krahn et al., 2009). Our results also show that PP2A methylation is important for its association with aPKCζ. This is significant since PP2A inhibits aPKCζ activity in MDCK cells (Nunbhakdi-Craig et al., 2002) and aPKCζ phosphorylates many proteins, including Par3, ZO-1 and occludin, thereby regulating the formation of protein complexes essential for TJ assembly and epithelial polarization (Hong, 2018; Van Itallie and Anderson, 2018; Martin et al., 2021). Conversely, inhibition of aPKCζ prevented the targeting of PP2A-Bα to cell-cell junctions. The resulting accumulation of cytoplasmic ABαC could contribute to the reported dephosphorylation of both ZO-1 and occludin that occurs following aPKCζ inhibition in MDCK cells (Jain et al., 2011).

Lastly, we show that key intermediates of one-carbon metabolism can influence TJ assembly in MDCK cells, at least in part by modulating PP2A methylation. Incubation with SAM further accentuated the negative effects of LCMT1 while inhibiting PME1 counteracted the stimulatory effects of Hcy/HTL during formation of cell-cell contacts in MDCK cells. LCMT1-dependent PP2Ac methylation is boosted by SAM and inhibited by Hcy (Sontag et al., 2007), but elevated Hcy levels may also promote PP2Ac demethylation by activating PME1 via an unspecified mechanism (Zhang et al., 2008). Very little is known on the role of one-carbon metabolism in epithelial TJ regulation. One report has linked hyperhomocysteinemia with worsening of intestinal barrier disruption in uremic rats (Liang et al., 2018). These detrimental effects likely result from oxidative stress, protein modifications, and cytoxicity induced by prolonged elevation of Hcy (Skovierova et al., 2016). In contrast, our results show that short-term incubation with Hcy/HTL can induce PP2A demethylation and exert a positive effect on TJ formation.

In conclusion, our studies identify novel LCMT1, PME1, and PP2A methylation/demethylation-dependent regulatory mechanisms that are critical for TJ assembly, integrity and barrier function in MDCK cells. Our findings uncover a link between one-carbon metabolism, PP2A methylation state, and the regulation of major TJ and polarity proteins in MDCK cells. Deregulation of these processes is likely to have major functional implications since TJs and epithelial polarity play a critical role in kidney homeostasis, and the kidney is an important site for one-carbon metabolism (Ducker and Rabinowitz, 2017). Indeed, disturbances in one-carbon metabolism (Karmin and Siow, 2018), deregulation of PP2A (Shao et al., 2021) and TJ dysfunction (Denker and Sabath, 2011; Szaszi and Amoozadeh, 2014; Bhat et al., 2018) are all linked to various kidney diseases, including carcinomas.

## Data Availability

The original contributions presented in the study are included in the article/Supplementary Material, further inquiries can be directed to the corresponding author.
