# Supplementary material for: A Novel Role of PP2A Methylation in the Regulation of Tight Junction Assembly and Integrity
Source: Front Cell Dev Biol. 2022 Jul 13;10:911279. doi: 10.3389/fcell.2022.911279 (PMC9326217; doi:10.3389/fcell.2022.911279)
Supplement: Supplementary file 1 [file DataSheet1.PDF]

Supplementary Material

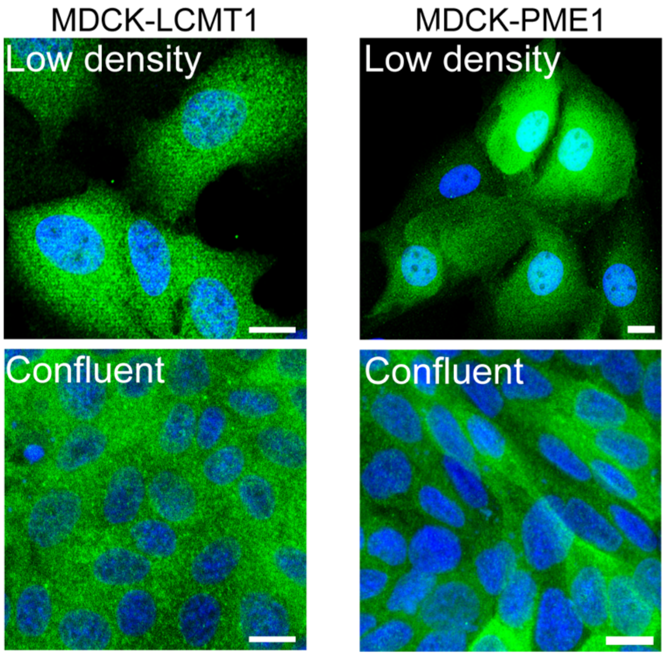

SUPPLEMENTARY FIGURE 1. Distribution of expressed LCMT1 and PME-1 in stable MDCK cell lines. Representative confocal images of LCMT1 (green) in MDCK-LCMT1 cells labeled with anti-HA antibodies, and PME1 (green) in MDCK-PME1 cells stained with anti-myc antibodies. Cell nuclei (blue) are stained with DAPI. Scale bars = 10 μm.

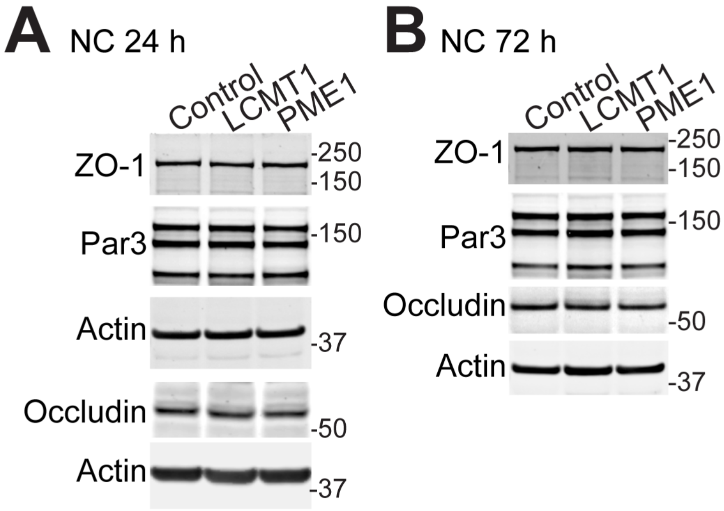

SUPPLEMENTARY FIGURE 2. Effects of overexpressed LCMT1 and PME1 on the protein expression levels of ZO-1, Par3 and occludin in MDCK cells. Representative Western blots of ZO-1, Par3 and occludin in total lysates from control MDCK, MDCK-LCMT1 and MDCK-PME-1 cells cultured for 24 h (**A**) or 72 h (**B**) in NC medium. Note that in MDCK cells, Par3 can migrate as three bands corresponding to Par3 splice variants. There were no statistically significant changes in the protein expression levels of the studied proteins among control MDCK, MDCK-LCMT1 and MDCK-PME1 cells, as determined after densitometry analyses of the immunoblots and normalization for protein loading ($n = 3$; $p > 0.05$).

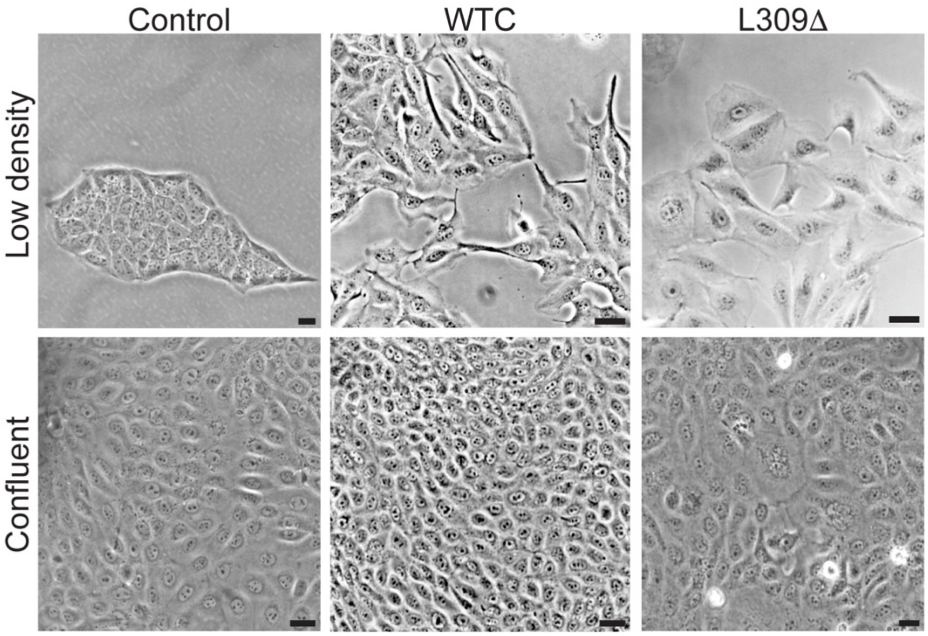

SUPPLEMENTARY FIGURE 3. Comparative analysis of control MDCK, MDCK-WTC and MDCK-L309Δ cell populations by phase contrast microscopy (20x objective). Representative images from three separate experiments are shown. Scale bars = 10 μm.

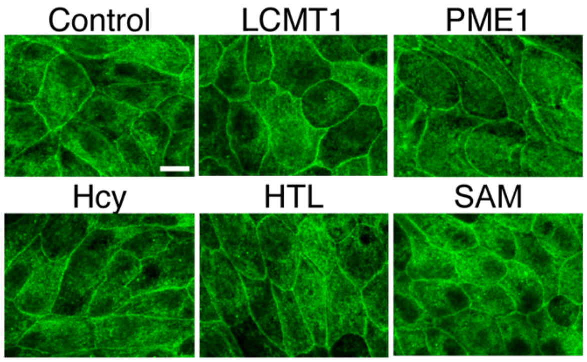

SUPPLEMENTARY FIGURE 4. Effects of LCMT1, PME1 and one-carbon metabolites on endogenous PP2A-Bα distribution in MDCK cells. Representative confocal images of the distribution of PP2A-Bα in control MDCK, MDCK-LCMT1 and MDCK-PME1 cells cultured in NC medium. A subset of control cells was treated overnight with either 100 μM Hcy, 100 μM HTL or 100 μM SAM in NC medium. Note that pools of PP2A-Bα are present at cell-cell junctions in all the cells examined. Scale bar = 10 μm.
